# Sex-Dependent Vascular Responses to Atorvastatin Across Multiple Arterial Beds in a Mouse Model of Marfan Syndrome

**DOI:** 10.3390/cells15131225

**Published:** 2026-07-07

**Authors:** Patrick Hunt, Kimberly Huynh, Brikena Gusek, Anna Stimpson, Roshanak Rahimian, Mitra Esfandiarei

**Affiliations:** 1 College of Osteopathic Medicine, Midwestern University, Glendale, AZ 85308, USA; 2 College of Graduate Studies, Midwestern University, Glendale, AZ 85308, USA; 3 Thomas J. Long School of Pharmacy, University of the Pacific, Stockton, CA 95211, USA; rrahimian@pacific.edu; 4 College of Medicine, University of Arizona, Phoenix, AZ 85004, USA; 5 Faculty of Medicine, University of British Columbia, Vancouver, BC V6T 1Z3, Canada

**Keywords:** Marfan syndrome, fibrillin-1, aortic aneurysm, cerebral artery, carotid artery, sex differences

## Abstract

Marfan syndrome (MFS) is characterized by progressive aortic aneurysm formation resulting from mutations in the fibrillin-1 (Fbn1) gene. Although the thoracic aorta is the primary site of pathology, accumulating evidence indicates that vascular dysfunction in MFS extends beyond the aorta to involve multiple arterial beds. Statins have been shown to attenuate aneurysm progression in experimental models of MFS; however, their effects on systemic vascular remodeling and arterial stiffness outside the aorta remain poorly characterized. In this study, we investigated the impact of chronic atorvastatin therapy on vascular structure and hemodynamic function across multiple vascular beds in the *Fbn1^C1041G/+^* mouse model of MFS. Male and female control and MFS mice received drinking water with or without atorvastatin (1 g/kg/day) from 4 weeks to 6 months of age, enabling the effects of atorvastatin to be assessed in both healthy and MFS arteries. High-frequency ultrasound imaging was used to assess vascular parameters in the aorta, left common carotid artery (LCCA), and posterior cerebral artery (PCA). Atorvastatin treatment significantly attenuated aortic root dilation in both male and female MFS mice and reduced aortic pulse wave velocity (PWV), indicating improved arterial compliance. In the carotid circulation, atorvastatin significantly reduced LCCA wall thickness and carotid PWV, although carotid wall strain did not improve. Atorvastatin raised both systolic and diastolic blood pressure in male and female MFS mice relative to untreated MFS animals, reaching levels not significantly different from untreated controls in both sexes, while having little effect in healthy controls apart from a rise in female diastolic pressure. In the posterior cerebral artery, peak systolic velocity, a hemodynamic index rather than a direct measure of perfusion, showed similarly sex-dependent changes, increasing in female MFS mice but decreasing further in males after atorvastatin. Collectively, these findings demonstrate that atorvastatin exerts systemic but heterogeneous vascular effects in MFS, improving arterial stiffness and structural remodeling across multiple arterial beds while producing sex-specific hemodynamic responses that warrant further investigation.

## 1. Introduction

Arterial stiffness and pathological vascular remodeling are major contributors to cardiovascular morbidity across a wide range of vascular diseases. These changes are particularly pronounced in connective tissue disorders such as Marfan syndrome (MFS), an autosomal dominant disorder caused by mutations in the fibrillin-1 (Fbn1) gene, which encodes a large extracellular matrix glycoprotein that serves as a structural scaffold for elastic fibers in multiple tissues, including the cardiovascular, ocular, and musculoskeletal systems [1]. Disruption of fibrillin-1 compromises microfibril integrity and leads to fragmentation of elastic fibers throughout the extracellular matrix [2]. MFS affects approximately 1 in 3000–5000 individuals worldwide. Although the incidence of the disorder is similar in men and women, males often develop more severe cardiovascular manifestations, including a higher incidence of ascending aortic aneurysm and a greater risk of dissection or rupture, the leading cause of mortality in MFS [3].

While the thoracic aorta is the most clinically recognized site of pathology in MFS, accumulating evidence indicates that vascular dysfunction in this condition is not confined to the aorta. Studies in experimental models have documented alterations in endothelial signaling, smooth muscle cell function, and vascular stiffness that affect systemic vascular physiology in MFS [4,5,6]. Our group recently demonstrated that phenotypic vascular dysfunction in the *Fbn1^C1041G/+^* mouse model extends beyond the aorta to include the carotid and cerebral circulations, with MFS mice exhibiting increased carotid wall thickness, elevated carotid pulse wave velocity, and reduced posterior cerebral artery blood flow velocity compared with wild-type controls [6]. These findings suggest that fibrillin-1 mutations produce widespread arteriopathy affecting structurally and functionally distinct vascular beds. Consistent with this, clinical observations in MFS patients have included reports of cerebrovascular complications such as intracranial aneurysm formation and stroke, though the prevalence and underlying mechanisms of cerebrovascular involvement in MFS remain incompletely defined [7,8,9]. Understanding how therapeutic interventions influence vascular function beyond the thoracic aorta is therefore important for determining whether treatments targeting aortic pathology also confer broader vascular protection.

Current pharmacological management of MFS primarily focuses on reducing hemodynamic stress on the aortic wall through β-blockers and angiotensin II type 1 receptor blockers (ARBs) [10]. Although these therapies can slow aortic root dilation, they do not fully prevent progressive vascular remodeling, and additional therapeutic strategies remain under investigation [11,12]. Statins, widely used as inhibitors of 3-hydroxy-3-methylglutaryl-coenzyme A (HMG-CoA) reductase, have attracted attention as potential modulators of vascular pathology beyond their well-known lipid-lowering effects. In addition to reducing circulating cholesterol, statins exert pleiotropic vascular effects, including modulation of endothelial function, inhibition of inflammatory signaling, and suppression of extracellular matrix remodeling through inhibition of matrix metalloproteinase secretion [13,14,15]. Previous studies have demonstrated that statins can attenuate aneurysm growth in mouse models of MFS [16,17]. In particular, Sato et al. showed that pravastatin reduced thoracic aortic aneurysm progression in *Fbn1* mutant mice through inhibition of Ras-dependent ERK signaling and a consequent reduction in matrix metalloproteinase activity [17]. While most studies of vascular pathology in MFS have focused on the thoracic aorta, emerging evidence indicates that alterations in arterial stiffness and vascular remodeling may also affect peripheral and cerebral circulation [6]. Understanding how therapeutic interventions influence these vascular beds is important for determining whether treatments targeting aortic pathology also improve broader vascular function.

Whether the vascular protection conferred by statins extends beyond the thoracic aorta to the peripheral and cerebral circulations affected in MFS has not been established. Therefore, we tested the hypothesis that chronic atorvastatin therapy improves vascular remodeling and arterial stiffness across multiple arterial beds in the *Fbn1^C1041G/+^* mouse model of MFS. Using high-frequency ultrasound imaging, we evaluated structural and hemodynamic parameters in the aorta, left common carotid artery, and posterior cerebral artery in male and female from both the control and MFS groups, with and without atorvastatin treatment, to determine whether atorvastatin produces systemic vascular effects beyond the thoracic aorta, whether those effects are uniform or vascular bed-specific, and whether they are specific to the MFS genotype or also occur in arteries from healthy control mice.

## 2. Materials and Methods

***Experimental Mouse Model:*** All animal procedures were conducted in accordance with the National Research Council Guide for the Care and Use of Laboratory Animals and were approved by the Institutional Animal Care and Use Committee (IACUC protocol AZ-4232). Animals were maintained on a 12 h light/dark cycle with ad libitum access to standard chow and water. MFS mice heterozygous for the *Fbn1* mutation associated with aortic root aneurysm and vascular dysfunction (*Fbn1^C1041G/+^*) were originally obtained from The Jackson Laboratory (Bar Harbor, ME, USA). The mutant line was backcrossed onto the *C57BL/6J* background for at least three generations to generate experimental animals and control littermates. Both male and female mice were used in the study. Animals were assigned to experimental groups at four weeks of age and distributed into eight experimental groups: untreated male control (n = 8), atorvastatin-treated male control (n = 8), untreated male MFS (n = 8), atorvastatin-treated male MFS (n = 8), untreated female control (n = 8), atorvastatin-treated female control (n = 8), untreated female MFS (n = 8), and atorvastatin-treated female MFS (n = 8). MFS mice at six months of age exhibit well-characterized vascular abnormalities including aortic root dilation, elastin fragmentation within the aortic wall, and increased aortic stiffness. Male and female mice were analyzed separately to evaluate potential sex-dependent differences in vascular phenotypes.

***Sample Size Calculation*:** An a priori power analysis was performed using data from our previously published study in the same MFS mouse model treated with losartan, in which aortic root diameter and aortic pulse wave velocity were measured. Because aortic root diameter exhibited greater variability than pulse wave velocity, this parameter was used as the primary endpoint for power analysis to provide a conservative estimate of the required sample size. Based on those data, the expected effect size was 0.47. Using a significance level of α = 0.05 and 80% statistical power, the calculated sample size required to detect treatment-related differences was 8 animals per group, which was used for all experimental groups in the present study.

***Atorvastatin Treatment:*** Atorvastatin was administered to male and female control and MFS mice to evaluate its potential therapeutic effects on vascular structure and function. Beginning at four weeks of age, mice received atorvastatin at a dose of 1 g/kg/day delivered through drinking water (refreshed every other day). This approach allowed continuous, non-invasive drug exposure throughout the duration of the study. The selected dose was based on previously published murine studies [17,18], and established interspecies dose conversion methods [19]. Because mice exhibit substantially higher metabolic rates than humans, and based on established allometric scaling principles, higher weight-adjusted doses are required to achieve comparable pharmacological exposure.

Drug concentration in the drinking water was calculated based on average body weight and estimated daily water consumption to ensure consistent dosing across animals. Water intake was monitored regularly throughout the treatment period, and no significant differences in water consumption were observed between experimental groups, indicating comparable drug exposure. Atorvastatin treatment was maintained continuously from 4 weeks to 6 months of age; the time point at which vascular phenotyping was performed.

***Body Weight & Blood Pressure Measurements:*** Body weight was recorded for all animals immediately prior to ultrasound imaging at six months of age using a calibrated digital balance. Measurements were obtained at the end of the treatment period on the same day as vascular phenotyping. Systolic and diastolic blood pressure measurements were obtained using a non-invasive tail-cuff system (CODA, Kent Scientific, Torrington, CT, USA). After an acclimation period, the occlusion cuff and volume-pressure recording cuff were positioned on the tail according to the manufacturer’s instructions. Animals underwent five acclimation cycles followed by fifteen measurement cycles. Acceptance of each cycle was determined automatically by the CODA software Version 4.2.2.0 based on signal quality; cycles that did not yield a valid volume-pressure signal were rejected by the system and excluded, and no manual selection was applied, minimizing operator-dependent variability. The first five successful cycles per animal were averaged for analysis. All measurements were performed under consistent environmental conditions and, when possible, at the same time of day to reduce circadian and handling-related variability.

***Ultrasound Imaging:*** Vascular structure and function were assessed using a Vevo2100 high-frequency ultrasound system (VisualSonics, FUJIFILM, Toronto, ON, Canada). Imaging was performed at six months of age using a transducer with a central frequency of 40 MHz, focal length of 7.0 mm, and frame rate of 557 frames per second. Mice were anesthetized with 3% isoflurane in 100% oxygen (2 L/min) in an induction chamber and maintained under anesthesia with 2% isoflurane delivered through a nose cone (1 L/min oxygen). Animals were positioned on a temperature-controlled platform equipped with electrocardiogram (ECG) monitoring, and body temperature was continuously monitored using a rectal probe. Adequate anesthesia was confirmed by loss of the toe-pinch reflex and righting response. Heart rate and respiratory rate were monitored throughout the procedure using ECG electrodes integrated into the heated platform. Hair was removed from the imaging sites (thorax and cranial region) using a depilatory cream to improve acoustic coupling. Ultrasound data were analyzed independently by three investigators blinded to sex, genotype, and treatment group, and final values were reported as the average of the three measurements.

### 2.1. Aortic Imaging and Pulse Wave Velocity Measurements

An MS550 transducer (VisualSonics, FUJIFILM, Toronto, ON, Canada) was used to obtain a five-mode aortic arch view for assessment of aortic root structure. Aortic root diameters were measured at the sinus of Valsalva in male and female MFS and control mice. Aortic stiffness was assessed by calculating pulse wave velocity (PWV). PW Doppler recordings were obtained from the ascending aorta, proximal to the brachiocephalic trunk, and from the descending aorta, distal to the left subclavian artery, using the aortic arch view. The aortic arch distance (mm) between these two measurement sites was determined using B-mode imaging along the central axis of the vessel. Transit time was defined as the time required for the pulse wave to travel between the proximal and distal Doppler measurement points. Pulse wave velocity was calculated as PWV = aortic arch distance/transit time.

### 2.2. Left Common Carotid Artery (LCCA) Measurements

Structural and functional properties of LCCA were evaluated by measuring wall thickness, arterial strain, and pulse wave velocity. LCCA wall thickness was determined using a short-axis M-mode view, with measurements obtained at three separate locations along the vessel and averaged. In the same imaging plane, systolic (Ds) and diastolic (Dd) luminal diameters were measured at three distinct points. Carotid arterial strain was calculated as Strain = (Ds − Dd)/Dd. Because this expression is a dimensionless ratio, the calculated strain is identical whether computed from vessel diameters or radii. Wall thickness was measured directly from M-mode images and was not derived from these diameter measurements, so the two parameters are independent. Carotid PWV was assessed using PW Doppler recordings obtained from two sites along the LCCA: a proximal site near the carotid bifurcation and a distal site approximately 5 mm upstream. The inter-site distance was measured using B-mode imaging, and transit time was determined from the foot-to-foot interval between the two Doppler waveforms. Carotid PWV was calculated using the same formula applied for aortic PWV: PWV = distance/transit time.

### 2.3. Posterior Cerebral Artery (PCA) Blood Flow Measurements

PCA blood flow was evaluated using Doppler ultrasound imaging. An MS250 transducer (VisualSonics, FUJIFILM, Toronto, ON, Canada) was used to obtain a coronal B-mode view of the mouse cranium. Color Doppler imaging was first applied to identify the posterior cerebral arteries, followed by pulse-wave Doppler measurements with adjustments to the Doppler angle and sampling volume to optimize flow detection. Five peak systolic velocity measurements were obtained from the Doppler waveform for each vessel and were averaged for analysis. PCA peak systolic velocity was used as a hemodynamic index; this measure reflects the combined influence of vascular resistance, vessel caliber, and upstream cardiac output, and should be interpreted accordingly.

***Statistical Analysis:*** All statistical analyses and graphical representations were performed using GraphPad Prism 11.01.1 software (GraphPad Software, San Diego, CA, USA). Measurements obtained from the three independent, blinded investigators were averaged prior to statistical analysis. To evaluate the effects of genotype and atorvastatin treatment within each sex, data were analyzed by two-way ANOVA with genotype (control vs. MFS) and treatment (untreated vs. atorvastatin) as the two independent variables. To evaluate sex differences in the response to atorvastatin, MFS and atorvastatin-treated MFS groups were compared by two-way ANOVA with sex (male vs. female) and treatment (untreated vs. atorvastatin) as the two independent variables. Tukey’s multiple comparisons test was applied for post hoc analysis when appropriate. Outliers were identified using the ROUT method (Q = 1%); no data points met the exclusion criterion, and all analyses were therefore performed on the complete dataset (n = 8 per group). Statistical significance was defined as *p* < 0.05.

## 3. Results

### 3.1. Atorvastatin Does Not Alter Body Weight in Control or MFS Mice

**Figure 1:**
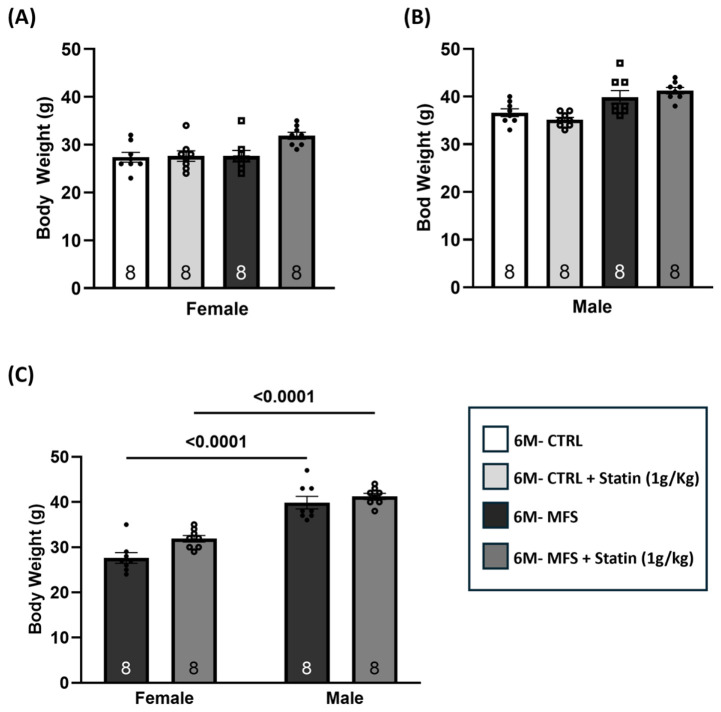
Atorvastatin does not alter body weight in control or MFS mice. (**A**) Body weight in female mice at six months of age. Atorvastatin did not significantly change body weight in either control or MFS females. (**B**) Body weight in male mice at six months of age. Atorvastatin did not significantly change body weight in either control or MFS males. (**C**) Between-sex comparison of body weight in the MFS and atorvastatin-treated MFS groups. Male mice weighed more than female mice in both groups (*p* < 0.0001). Data are presented as mean ± SEM; n = 8 animals per group. Within-sex comparisons (**A**,**B**) were analyzed by two-way ANOVA with genotype and treatment as factors, and the between-sex comparison (**C**) by two-way ANOVA with sex and treatment as factors, each followed by Tukey’s multiple comparisons test. Exact *p*-values are shown on the graphs for all significant comparisons (*p* < 0.05).

To determine whether atorvastatin influences body weight, treated animals were compared with untreated animals of the same genotype. Atorvastatin did not significantly affect body weight in control or MFS mice of either sex (Figure 1A,B). As expected, male mice weighed significantly more than female mice in both the MFS and atorvastatin-treated MFS groups (both *p* < 0.0001; Figure 1C).

### 3.2. Atorvastatin Increases Blood Pressure (BP) in Male and Female MFS Mice

**Figure 2:**
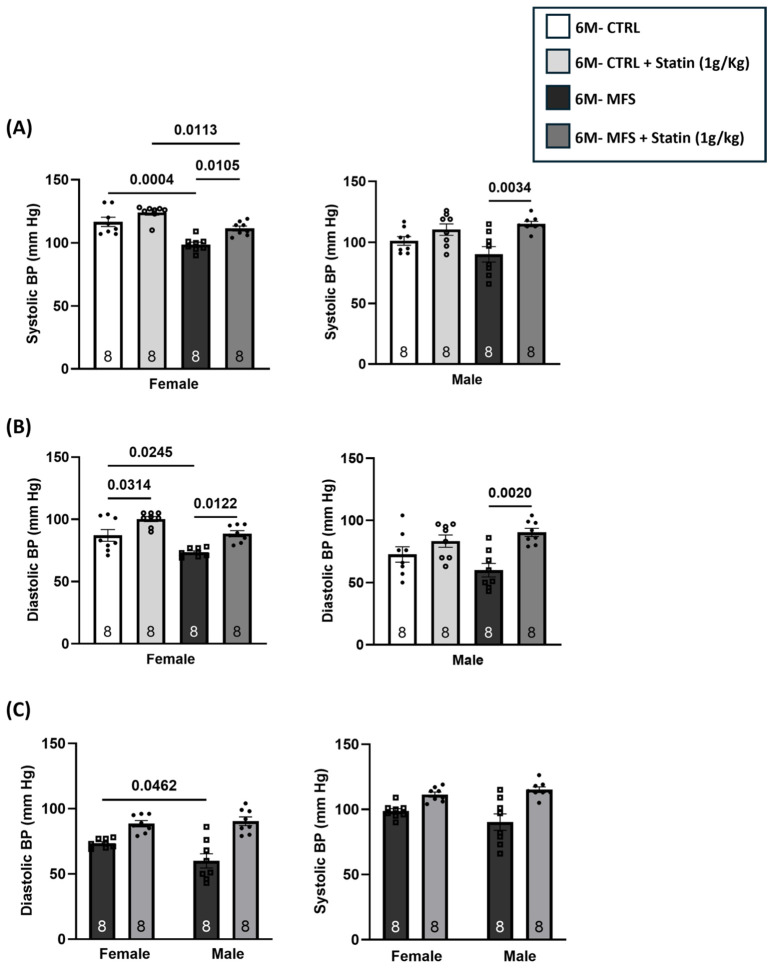
Atorvastatin increases blood pressure in male and female MFS mice. (**A**) Systolic blood pressure in female and male mice at six months of age. Female MFS mice had lower systolic pressure than female control mice (*p* = 0.0004), and atorvastatin raised systolic pressure in female MFS mice relative to untreated MFS animals (*p* = 0.0105); treated female MFS mice remained below atorvastatin-treated female control mice (*p* = 0.0113). In males, atorvastatin raised systolic pressure in MFS mice relative to untreated MFS animals (*p* = 0.0034), to values not different from either control group. (**B**) Diastolic blood pressure in female and male mice. Female MFS mice had lower diastolic pressure than female control mice (*p* = 0.0245), and atorvastatin raised diastolic pressure in female MFS mice relative to untreated MFS animals (*p* = 0.0122). Atorvastatin also raised diastolic pressure in female control mice relative to untreated female controls (*p* = 0.0314). In males, atorvastatin raised diastolic pressure in MFS mice relative to untreated MFS animals (*p* = 0.0020). (**C**) Between-sex comparison of systolic and diastolic blood pressure. Untreated female MFS mice had higher diastolic pressure than untreated male MFS mice (*p* = 0.0478); no sex difference was observed for systolic pressure or between the atorvastatin-treated groups. Data are presented as mean ± SEM; n = 8 animals per group. Within-sex comparisons (**A**,**B**) were analyzed by two-way ANOVA with genotype and treatment as factors, and the between-sex comparison (**C**) by two-way ANOVA with sex and treatment as factors, each followed by Tukey’s multiple comparisons test. Exact *p*-values are shown on the graphs for all significant comparisons (*p* < 0.05).

Systolic and diastolic blood pressure were measured at six months of age using non-invasive tail-cuff plethysmography. In females, MFS mice showed significantly lower systolic BP than untreated control (*p* = 0.0004, Figure 2A). Atorvastatin significantly increased systolic BP in MFS females relative to untreated MFS animals (*p* = 0.0105, Figure 2A), reaching a level not significantly different from untreated control. It is noteworthy that in statin-treated MFS females the systolic BP remained lower than atorvastatin-treated control females (*p* = 0.0113; Figure 2A). In males, atorvastatin significantly increased systolic pressure in MFS mice relative to sex-matched untreated MFS(*p* = 0.0034, Figure 2A), reaching a level not significantly different from either control group (Figure 2A).

Diastolic blood pressure followed a similar pattern. Female MFS mice displayed significantly lower diastolic BP compared with female controls (*p* = 0.0245; Figure 2B), whereas atorvastatin treatment significantly increased diastolic BP relative to untreated female MFS mice (*p* = 0.0122, Figure 2B), restoring values to within the control range. Diastolic BP in untreated male MFS mice was not significantly different from sex-matched controls; however, in male MFS mice, atorvastatin treatment significantly increased diastolic blood pressure relative to untreated MFS subjects (*p* = 0.0020; Figure 2B).

Comparison of diastolic and systolic BP between male and female MFS groups demonstrated that diastolic BP in male MFS mice was significantly lower compared to female MFS mice (*p* = 0.0478, Figure 2C) with no differences observed between statin-treated MFS male and female groups (Figure 2C). There were no significant differences in systolic BP between male and female groups (Figure 2C).

### 3.3. Atorvastatin Attenuates Aortic Root Enlargement in Male and Female MFS Mice

**Figure 3:**
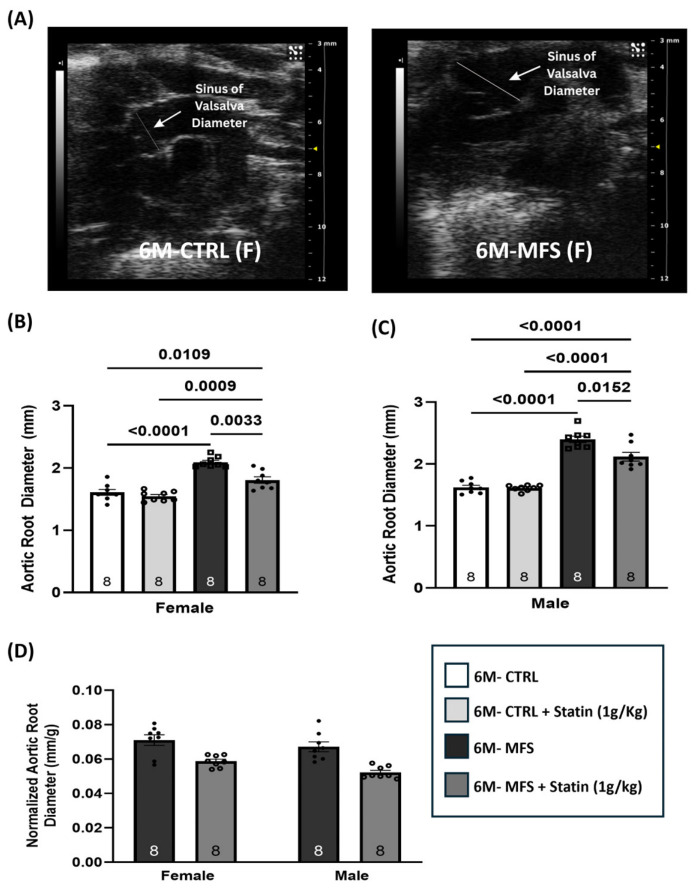
Atorvastatin attenuates aortic root enlargement in male and female MFS mice. (**A**) Representative high-frequency ultrasound images of the aortic root from 6-month-old control and MFS mice. (**B**) Aortic root diameter in female mice. MFS females had larger aortic roots than untreated (*p* < 0.0001) and atorvastatin-treated (*p* = 0.0009) controls, and atorvastatin reduced aortic root diameter relative to untreated MFS animals (*p* = 0.0033); treated MFS females remained larger than control (*p* = 0.0109), indicating a partial reduction. (**C**) Aortic root diameter in male mice. MFS males had larger aortic roots than controls (*p* < 0.0001), atorvastatin reduced diameter relative to untreated MFS animals (*p* = 0.0152), and treated MFS males remained larger than both untreated (*p* < 0.0001) and atorvastatin-treated (*p* < 0.0001) controls. (**D**) Between-sex comparison of body weight-normalized aortic root diameter, which did not differ between males and females. Data are presented as mean ± SEM; n = 8 animals per group. Within-sex comparisons (**B**,**C**) were analyzed by two-way ANOVA with genotype and treatment as factors, and the between-sex comparison (**D**) by two-way ANOVA with sex and treatment as factors, each followed by Tukey’s multiple comparisons test. Exact *p*-values are shown on the graphs for all significant comparisons (*p* < 0.05).

Aortic root diameter was measured at the sinus of Valsalva using high-frequency ultrasound in CTRL, MFS, and atorvastatin-treated CTRL and MFS mice at six months of age (Figure 3A). As expected, non-treated female and male MFS mice exhibited significantly greater aortic root diameter compared with sex-matched non-treated controls (*p* < 0.0001; Figure 3 B,C). Atorvastatin treatment significantly reduced aortic root diameter in female (*p* = 0.0033, Figure 3B) and male (*p* = 0.0152, Figure 3C) MFS mice relative to untreated sex-matched MFS mice, with no difference observed in male and female CTRL groups. It is important to note that in both statin-treated male and female MFS mice, aortic root diameters were still significantly larger than untreated sex- and age-matched CTRL mice, indicating a partial reduction (Figure 3B,C). Because male mice weigh more than females and aortic root diameter scales with body size, between-sex comparisons were performed using body-weight-normalized values; normalized aortic root diameter did not differ significantly between males and females (Figure 3D).

### 3.4. Atorvastatin Reduces Aortic Wall Stiffness (Pulse Wave Velocity) in MFS Mice

**Figure 4:**
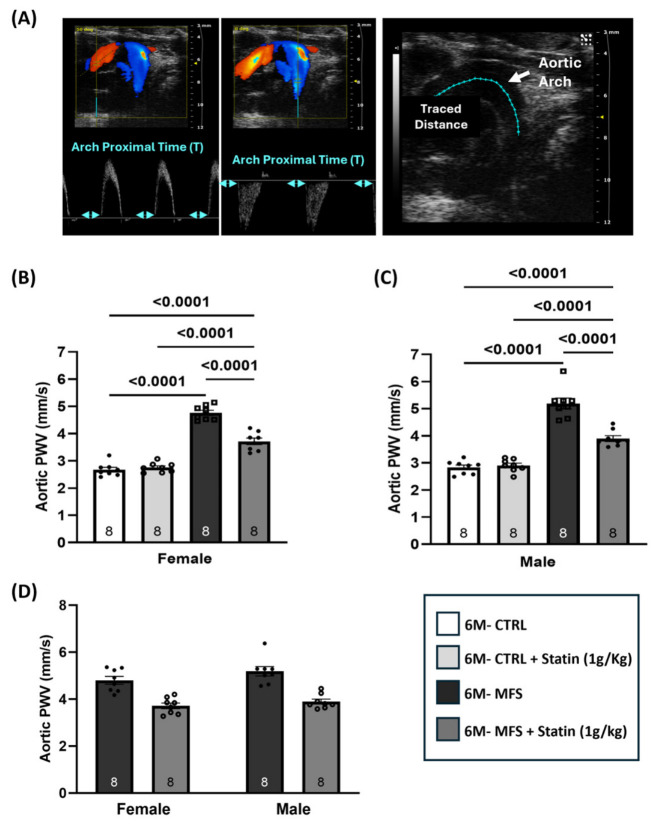
Atorvastatin reduces aortic wall stiffness (pulse wave velocity) in MFS mice. (**A**) Representative Doppler ultrasound recordings used to calculate aortic pulse wave velocity (PWV). (**B**) Aortic PWV in female mice. PWV was higher in MFS females than in untreated and atorvastatin-treated controls (both *p* < 0.0001); atorvastatin reduced PWV relative to untreated MFS animals (*p* < 0.0001) but treated values remained above control (*p* < 0.0001), indicating a partial reduction. (**C**) Aortic PWV in male mice, showing the same pattern: elevated PWV in MFS (*p* < 0.0001), reduction with atorvastatin (*p* < 0.0001), and treated values remaining above control (*p* < 0.0001). (**D**) Between-sex comparison of aortic PWV, which did not differ between males and females. Data are presented as mean ± SEM; n = 8 animals per group. Within-sex comparisons (**B**,**C**) were analyzed by two-way ANOVA with genotype and treatment as factors, and the between-sex comparison (**D**) by two-way ANOVA with sex and treatment as factors, each followed by Tukey’s multiple comparisons test. Exact *p*-values are shown on the graphs for all significant comparisons (*p* < 0.05).

Aortic pulse wave velocity (PWV) was measured as an indicator of arterial stiffness (Figure 4A). In female mice, MFS significantly increased aortic PWV compared with controls (*p* < 0.0001; Figure 4B). Atorvastatin treatment significantly reduced PWV in female MFS mice relative to untreated female MFS mice (*p* < 0.0001, Figure 4B), although values remained elevated compared with untreated and treated controls (*p* < 0.0001, Figure 4B), indicating partial reduction. Male MFS mice also exhibited significantly increased aortic PWV compared with male controls (*p* < 0.0001; Figure 4C). In males, atorvastatin treatment significantly reduced PWV (*p* < 0.0001; Figure 4C) but did not fully restore values to untreated and treated control levels (*p* < 0.0001, Figure 4C), which again is an indication of partial reduction. No significant sex differences in aortic PWV were observed between untreated or atorvastatin-treated MFS groups (Figure 4D).

### 3.5. Atorvastatin Normalizes Left Common Carotid Artery Wall Thickness in Male and Female MFS Mice

**Figure 5:**
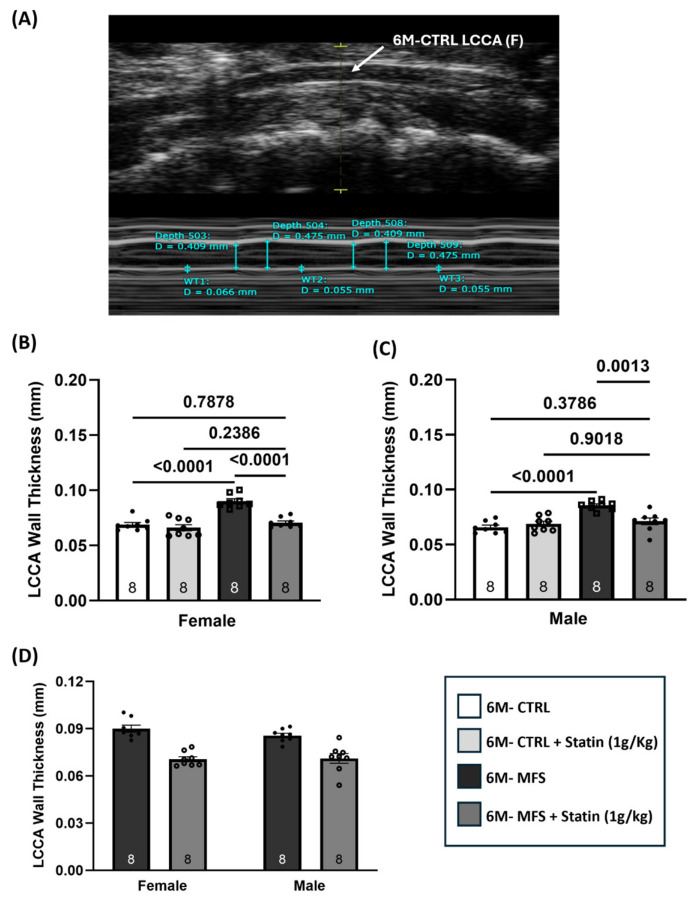
Atorvastatin normalizes left common carotid artery wall thickness in male and female MFS mice. (**A**) Representative ultrasound image of the left common carotid artery (LCCA) used to measure wall thickness. (**B**) LCCA wall thickness in female mice. Wall thickness was greater in MFS females than controls (*p* < 0.0001) and was reduced by atorvastatin relative to untreated MFS animals (*p* < 0.0001), to values not different from untreated (*p* = 0.7878) or atorvastatin-treated (*p* = 0.2386) controls. (**C**) LCCA wall thickness in male mice. Wall thickness was greater in MFS males than controls (*p* < 0.0001) and was reduced by atorvastatin (*p* = 0.0013), to values not different from untreated (*p* = 0.3786) or atorvastatin-treated (*p* = 0.9018) controls. In both sexes, wall thickness in treated MFS mice was fully normalized. (**D**) Between-sex comparison of carotid wall thickness, which did not differ between males and females. Data are presented as mean ± SEM; n = 8 animals per group. Within-sex comparisons (**B**,**C**) were analyzed by two-way ANOVA with genotype and treatment as factors, and the between-sex comparison (**D**) by two-way ANOVA with sex and treatment as factors, each followed by Tukey’s multiple comparisons test. Exact *p*-values are shown on the graphs for all significant comparisons (*p* < 0.05).

Structural remodeling of the carotid artery was assessed by LCCA wall thickness (Figure 5A). Female MFS mice displayed significantly greater LCCA wall thickness compared with female controls (*p* < 0.0001; Figure 5B). Atorvastatin treatment significantly reduced carotid wall thickness in female MFS mice relative to untreated MFS animals (*p* < 0.0001). Male MFS mice also exhibited significantly increased LCCA wall thickness compared with male controls (*p* < 0.0001; Figure 5C). Atorvastatin treatment significantly reduced wall thickness in male MFS mice (*p* = 0.0013, Figure 5C). In both sexes, LCCA wall thickness in atorvastatin-treated MFS mice was no longer significantly different from control values, confirming full normalization in both sexes (Figure 5B,C). No significant sex differences were observed between untreated or atorvastatin-treated MFS groups (Figure 5D).

### 3.6. Atorvastatin Does Not Restore Left Common Carotid Artery Wall Strain

**Figure 6:**
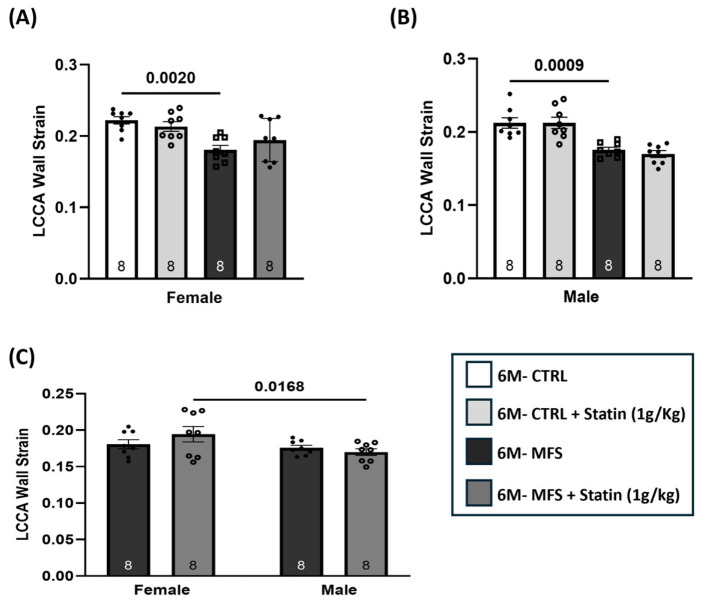
Atorvastatin does not restore left common carotid artery wall strain. (**A**) Carotid wall strain in MFS females was lower than controls (*p* = 0.0020); atorvastatin did not significantly change strain relative to untreated MFS animals. (**B**) Carotid wall strain in MFS males was lower than controls (*p* = 0.0009); atorvastatin did not improve strain relative to untreated MFS animals (*p* = 0.8831). (**C**) Atorvastatin-treated female MFS mice retained higher strain than atorvastatin-treated male MFS mice (*p* = 0.0168). Data are presented as mean ± SEM; n = 8 animals per group. Within-sex comparisons (**A**,**B**) were analyzed by two-way ANOVA with genotype and treatment as factors, and the between-sex comparison (**C**) by two-way ANOVA with sex and treatment as factors, each followed by Tukey’s multiple comparisons test. Exact *p*-values are shown on the graphs for all significant comparisons (*p* < 0.05).

LCCA wall strain was calculated from systolic and diastolic luminal diameters at six months of age. In females, wall strain was lower in MFS mice than in control mice (*p* = 0.0020, Figure 6A). Atorvastatin did not change strain in MFS females relative to untreated MFS mice (Figure 6A). In males, wall strain was lower in MFS than in control mice (*p* = 0.0009, Figure 6B). Atorvastatin did not improve LCCA wall strain in male MFS mice (*p* = 0.8831, Figure 6B). Between sexes, atorvastatin-treated female MFS mice retained higher wall strain than atorvastatin-treated male MFS mice (*p* = 0.0168; Figure 6C).

### 3.7. Atorvastatin Reduces Carotid Wall Stiffness (Pulse Wave Velocity) in MFS Mice

**Figure 7:**
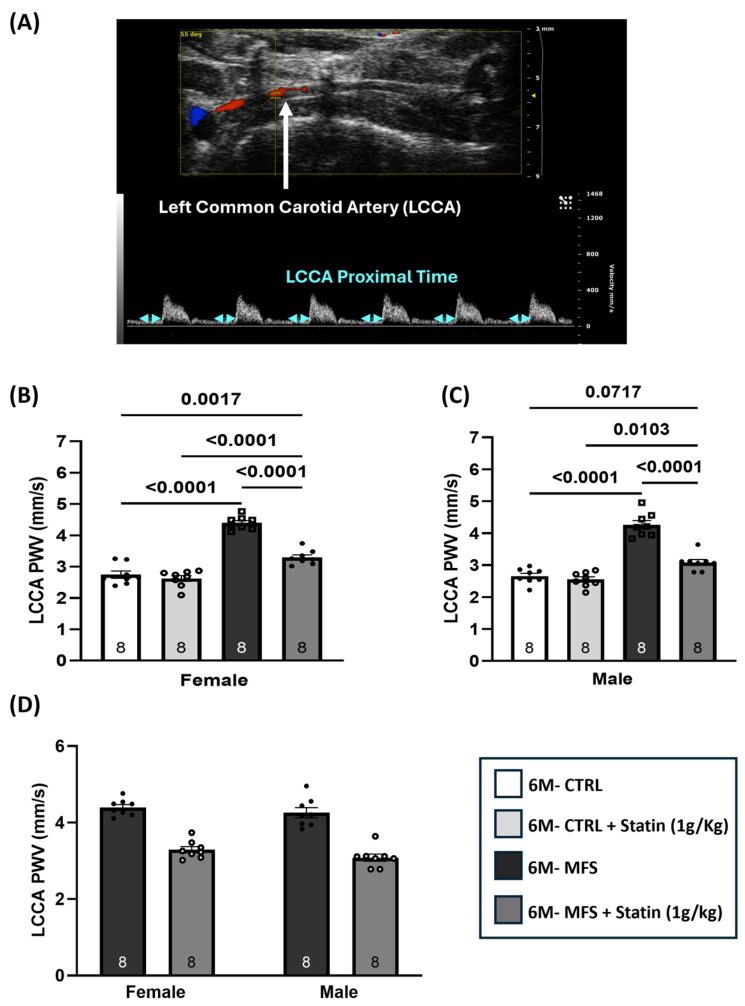
Atorvastatin reduces carotid wall stiffness (pulse wave velocity) in MFS mice. (**A**) Representative Doppler ultrasound recordings used to calculate carotid PWV. (**B**) Carotid PWV in female mice. PWV was higher in MFS females than untreated and atorvastatin-treated controls (both *p* < 0.0001); atorvastatin reduced PWV relative to untreated MFS animals (*p* < 0.0001) but treated values remained above control (*p* = 0.0017), indicating a partial reduction. (**C**) Carotid PWV in male mice. PWV was higher in MFS males than controls (*p* < 0.0001) and was reduced by atorvastatin relative to untreated MFS animals (*p* < 0.0001). Treated MFS males were not different from untreated control (*p* = 0.0717) but remained higher than atorvastatin-treated control (*p* = 0.0103). (**D**) Between-sex comparison of carotid PWV, which did not differ between males and females. Data are presented as mean ± SEM; n = 8 animals per group. Within-sex comparisons (**B**,**C**) were analyzed by two-way ANOVA with genotype and treatment as factors, and the between-sex comparison (**D**) by two-way ANOVA with sex and treatment as factors, each followed by Tukey’s multiple comparisons test. Exact *p*-values are shown on the graphs for all significant comparisons (*p* < 0.05).

Carotid PWV was measured to determine whether atorvastatin treatment alters carotid arterial stiffness (Figure 7A). Female MFS mice exhibited significantly higher carotid PWV compared with controls (*p* < 0.0001; Figure 7B). Atorvastatin treatment significantly reduced carotid PWV in female MFS mice (*p* < 0.0001, Figure 7B). In female MFS groups, PWV values in atorvastatin-treated MFS mice were still significantly higher compared to both non-treated (*p* = 0.0017, Figure 7B) and statin-treated female CTRL groups (*p* < 0.0001; Figure 7B), indicating a partial correction of carotid wall stiffness. In males, carotid PWV was higher in MFS than in control mice (*p* < 0.0001, Figure 7C), and treatment with atorvastatin significantly reduced carotid PWV in male MFS mice (*p* < 0.0001; Figure 7C). Treated MFS males were not significantly different from untreated control mice (*p* = 0.0717, Figure 7C) but remained higher than atorvastatin-treated control mice (*p* = 0.0103, Figure 7C). Carotid PWV did not differ significantly between males and females (Figure 7D).

### 3.8. Posterior Cerebral Artery Blood Flow Velocity Responds to Atorvastatin in a Sex-Dependent Manner

**Figure 8:**
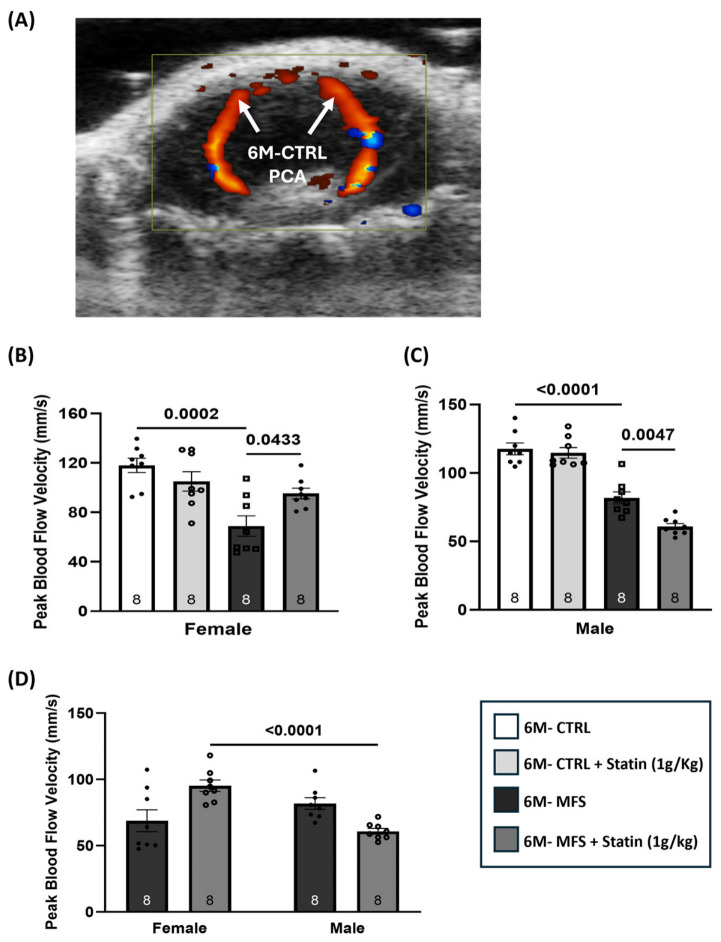
Posterior cerebral artery blood flow velocity shows sex-dependent responses to atorvastatin. (**A**) Representative ultrasound image showing the posterior cerebral artery (PCA) used for Doppler measurements. (**B**) PCA peak systolic velocity in female mice. Peak velocity was lower in MFS females than controls (*p* = 0.0002), and atorvastatin increased peak velocity relative to untreated MFS animals (*p* = 0.0433), toward control levels. (**C**) PCA peak systolic velocity in male mice. Peak velocity was lower in MFS males than controls (*p* < 0.0001), and atorvastatin further reduced peak velocity relative to untreated MFS animals (*p* = 0.0047). (**D**) Between-sex comparison of PCA peak systolic velocity. Atorvastatin-treated female MFS mice had higher peak velocity than atorvastatin-treated male MFS mice (*p* < 0.0001). Data are presented as mean ± SEM; n = 8 animals per group. Within-sex comparisons (**B**,**C**) were analyzed by two-way ANOVA with genotype and treatment as factors, and the between-sex comparison (**D**) by two-way ANOVA with sex and treatment as factors, each followed by Tukey’s multiple comparisons test. Exact *p*-values are shown on the graphs for all significant comparisons (*p* < 0.05).

Peak systolic blood flow velocity in the PCA was assessed using ultrasound imaging (Figure 8A). Female MFS mice exhibited significantly lower PCA peak velocity compared with female controls (*p* = 0.0002; Figure 8B). Atorvastatin treatment significantly increased PCA peak velocity in female MFS mice compared with untreated MFS mice, restoring values toward control levels (*p* = 0.0433, Figure 8B). In male mice, PCA peak velocity was also reduced in MFS animals compared with controls (*p* < 0.0001; Figure 8C). In contrast to the response observed in females, atorvastatin treatment further reduced PCA peak velocity in male MFS mice relative to untreated MFS mice (*p* = 0.0047, Figure 8C). Direct comparison between sexes confirmed that PCA peak velocity was significantly higher in atorvastatin-treated female MFS mice than in atorvastatin-treated males (*p* < 0.0001; Figure 8D), underscoring the sex-dependent nature of the cerebrovascular response to atorvastatin in this model.

## 4. Discussion

In this study, we evaluated the effects of long-term atorvastatin treatment on vascular remodeling and hemodynamic function across multiple arterial beds in the *Fbn1^C1041G/+^* mouse model of MFS. The principal findings are as follows: atorvastatin attenuated aortic root dilation and reduced arterial stiffness, as reflected by lower PWV in both the aorta and carotid artery; structural remodeling of the carotid artery was improved, with significant reductions in wall thickness, and these beneficial effects extended beyond the thoracic aorta to the peripheral vasculature. At the same time, atorvastatin raised blood pressure in MFS mice of both sexes to levels comparable with those of healthy controls, elicited opposing changes in cerebral blood flow velocity between male and female MFS mice, and had no effect on carotid wall strain. Taken together, these findings indicate that statins may exert systemic but heterogeneous vascular effects across multiple arterial beds in MFS, and that sex is an important modifier of the hemodynamic response to atorvastatin in this model.

The attenuation of aortic root dilation observed in this study is consistent with prior work demonstrating beneficial effects of statins in experimental MFS. McLoughlin et al. reported that pravastatin reduced aortic root enlargement in MFS mice [16]. Sato et al. demonstrated that pravastatin suppressed thoracic aortic aneurysm progression in *Fbn1* mutant mice through inhibition of Ras-dependent ERK signaling and a consequent reduction in matrix metalloproteinase (MMP) activity [17]. The present findings extend these observations to atorvastatin and confirm that statin-mediated attenuation of aortic root dilation is reproducible across different statins in this model. It should be noted, however, that atorvastatin treatment reduced but did not normalize aortic root diameter in either sex, as values in treated MFS mice remained significantly elevated above controls, indicating that atorvastatin partially attenuates, rather than fully prevents, aortic root pathology at the dose and treatment duration used here. It is important to highlight that this observation is consistent with the clinical management of Marfan syndrome, where the primary goal of pharmacological therapy is generally to slow aneurysm progression and delay surgical intervention rather than restore normal aortic dimensions. In this context, the magnitude of aortic root reduction observed in the present study (estimated 13.6% reduction in female MFS mice and 11.6% reduction in male MFS mice) is likely to be biologically meaningful despite the persistence of residual dilation. Whether higher doses, earlier intervention, or combination therapy could achieve fuller normalization remains an open question.

Elevated PWV is a well-recognized feature of MFS-associated vasculopathy and has been documented in both experimental models and clinical cohorts [3,6]. The reduction in aortic PWV following atorvastatin treatment (estimated 22.7% reduction in female MFS mice and 25% reduction in male MFS mice) suggests improved mechanical properties of the aortic wall, consistent with attenuation of ongoing remodeling. Statins have been shown to suppress MMP-mediated extracellular matrix degradation and reduce vascular smooth muscle cell proliferation and migration through inhibition of the Rho/ROCK pathway [14,17,20]; either or both of these mechanisms could plausibly contribute to improved aortic wall mechanics. Atorvastatin reduced aortic and carotid PWV even though it raised blood pressure in treated MFS mice, indicating that the improvement in arterial stiffness was not a secondary consequence of blood pressure lowering. This is consistent with prior observations that statin-associated improvements in arterial stiffness can be at least partly blood pressure-independent [21,22].

Beyond the aorta, atorvastatin treatment significantly reduced LCCA wall thickness and carotid PWV in both male and female MFS mice. Carotid wall thickness was fully normalized, returning to values that did not differ from either untreated or atorvastatin-treated control mice (estimated 22.2% reduction in female MFS mice and estimated 16.5% reduction in male MFS mice). However, like aorta, atorvastatin markedly reduced carotid PWV (estimated 25% reduction in female MFS and 27.6% reduction in male MFS mice) to levels that were still above statin-treated control levels. The complete normalization of carotid wall thickness thus contrasts with the partial reduction seen for aortic root diameter and for pulse wave velocity in both arterial beds. This difference may reflect the distinct structural composition of these vascular beds. The thoracic aorta is a highly elastic artery characterized by extensive elastin lamellae that undergo progressive fragmentation in MFS; once structural elastin is lost, pharmacological intervention is unlikely to reverse this damage. The carotid artery, by contrast, has a relatively higher proportion of vascular smooth muscle cells and a greater dependence on cellular remodeling mechanisms, including VSMC proliferation, migration, and collagen deposition, for changes in wall thickness [23,24]. These processes are more amenable to statin-mediated suppression via Rho/ROCK inhibition and MMP downregulation than is elastin repair [14,20], which may explain why the carotid wall thickness normalized fully while aortic root dilation did not. This interpretation is mechanistically plausible but was not directly tested in the current study; histological assessment of elastin integrity, collagen content, and MMP activity in both vascular beds would be required to confirm it.

Despite the improvements in carotid wall thickness and PWV, atorvastatin treatment did not restore carotid wall strain in either sex. This dissociation between PWV and wall strain warrants careful interpretation. Although both parameters are commonly used as indicators of arterial stiffness, they reflect distinct biomechanical properties and do not necessarily change in parallel. PWV represents the propagation velocity of pressure waves along the arterial tree and is influenced by both the intrinsic elastic modulus of the wall and vascular smooth muscle tone; wall strain, in contrast, reflects local vessel distensibility during the cardiac cycle and is more sensitive to the residual structural integrity of the elastic network within the wall [25,26,27].

If the primary mechanism of atorvastatin action in MFS is suppression of ongoing cellular remodeling and MMP-mediated matrix degradation, rather than restoration of elastin structure, one would predict improvement in PWV without proportional recovery of local wall strain, since the latter depends on the integrity of elastic fibers that are already fragmented. This mechanistic distinction is supported by evidence that statins reduce MMP secretion from vascular smooth muscle cells and macrophages [14,20] and inhibit Rho/ROCK-mediated cytoskeletal tension, both of which would be expected to lower PWV by reducing wall stiffness driven by cellular and matrix remodeling, while leaving the underlying elastic fiber deficit unaddressed [28,29]. This remains a plausible rather than a proven explanation, as direct histological evidence of elastin integrity in these animals was not obtained.

The blood pressure findings in this study warrant careful interpretation, particularly given the inherent variability associated with tail-cuff plethysmography. Atorvastatin raised both systolic and diastolic blood pressure in MFS mice of both sexes, from the reduced values observed in untreated MFS animals to levels that did not differ from those of healthy control mice. This is broadly concordant with prior observations that statin-induced improvements in endothelial nitric oxide signaling, and vascular tone can modestly reduce blood pressure under pathological cardiovascular conditions [28,29]. Notably, statins exert little or no effect on blood pressure in normotensive wild-type animals, and any blood pressure changes observed with statin therapy are generally regarded as secondary pleiotropic effects that are more pronounced under conditions of existing vascular dysfunction [30,31,32]. Our data are consistent with this interpretation: atorvastatin had no significant effect on blood pressure in healthy control mice of either sex, with the single exception of an increase in diastolic pressure in treated female controls. The inclusion of atorvastatin-treated control animals therefore indicates that the blood pressure response to atorvastatin in this model depends largely on the MFS disease context rather than reflecting a generalized pressor action of the drug.

The cerebral circulation displayed the most complex and sex-divergent responses to atorvastatin in this study. PCA peak systolic velocity was reduced in MFS mice of both sexes compared with controls, consistent with our previously published observation of cerebrovascular dysfunction in this model [6]. In females, atorvastatin treatment increased PCA peak velocity toward control levels (estimated 27.7% increase), whereas in males it produced a further reduction below already-reduced MFS value (estimated 25.6% decrease). Before mechanistic interpretation, it is important to acknowledge an inherent limitation of this endpoint: PCA peak systolic velocity is a Doppler-derived measure that reflects the combined influence of vascular resistance, vessel caliber, cardiac output, and heart rate. An increase in peak velocity could represent vasodilation and improved cerebral perfusion, or alternatively, increased cardiac output; conversely, a decrease could reflect vasoconstriction, a reduced cardiac output, or increased downstream resistance. These possibilities cannot be distinguished with peak velocity measurements alone, and vascular resistance indices such as the resistance index or pulsatility index would be needed to differentiate them. With this caveat clearly stated, the divergent PCA response between sexes is among the clearest sex-dependent effects of atorvastatin in this study, and it cannot be attributed to a parallel divergence in blood pressure, since treated male and female MFS mice reached comparable blood pressure levels.

Mechanistically, sex-specific differences in cerebrovascular regulation have been documented and may involve hormonal modulation of endothelial signaling pathways [33,34,35]. Estrogen has been shown to enhance eNOS activity and nitric oxide bioavailability, and statins can further potentiate nitric oxide signaling through Rho/ROCK inhibition and PI3K/Akt activation [28,29,36,37]. In female MFS mice, the combined effect of endogenous estrogenic signaling and statin-enhanced nitric oxide production may promote cerebrovascular vasodilation, contributing to the increase in PCA peak velocity observed. In males, the absence of this estrogenic augmentation may contribute to the net reduction in peak flow velocity, although the responsible mechanism cannot be determined from the present data. This is a plausible framework consistent with the available data, but it is speculative and was not directly tested. Future studies incorporating cerebrovascular resistance measurements, eNOS expression analysis, and sex hormone profiling would be needed to evaluate this hypothesis rigorously.

Collectively, the findings of this study support the conclusion that long-term atorvastatin treatment produces systemic vascular benefits in MFS that extend beyond the thoracic aorta, including improvements in carotid wall structure and arterial stiffness across multiple vascular beds. The heterogeneity of responses across vascular beds and between sexes highlights the importance of examining treatment effects beyond the aorta and of analyzing male and female animals separately in preclinical MFS research. Whether the vascular improvements observed here translate to reduced cardiovascular risk in MFS patients cannot be determined from this preclinical study, and the clinical relevance of the murine dose used remains an important consideration. These findings nonetheless provide a rationale for further investigation of statins as adjunctive therapy for the broader vasculopathy associated with MFS, particularly in the context of sex-stratified analysis.

## 5. Limitations

Several limitations of the present study should be considered. First, vascular assessments were based on high-frequency ultrasound measurements, which provide functional evaluation of arterial structure and hemodynamics but do not directly assess histological changes within the vessel wall. The mechanistic arguments advanced in this discussion, particularly those invoking MMP-mediated matrix degradation, elastin fragmentation, and Rho/ROCK-dependent VSMC remodeling as the basis for differential responses across vascular beds, mainly rely on plausible biological reasoning supported by prior literature rather than direct histological evidence from the animals studied here. Future studies incorporating Verhoeff-Van Gieson staining for elastin integrity, Masson’s trichrome for collagen deposition, and immunohistochemical quantification of MMP expression and activity in aortic and carotid tissue will be necessary to determine whether the functional improvements observed with atorvastatin treatment correspond to measurable changes in extracellular matrix composition and vascular wall architecture.

Second, the present study focused on physiological and hemodynamic outcomes and did not investigate the molecular mechanisms underlying the observed vascular responses. In particular, the sex-dependent effect of atorvastatin on cerebral blood flow velocity raises questions about potential differences in eNOS signaling and Rho/ROCK pathway activity between male and female MFS mice that were not addressed here. Molecular profiling of these pathways in both sexes, alongside sex hormone measurements, would be required to evaluate the mechanistic hypotheses proposed in this discussion.

Third, all vascular assessments were performed at a single time point (six months of age) following continuous treatment from four weeks of age. This design does not permit determination of when during the treatment course the observed vascular effects emerge, nor can it distinguish whether atorvastatin is preventing disease progression or partially reversing established pathology. Longitudinal studies with intermediate assessment time points are needed to address these questions.

Finally, although the sample size was sufficient to detect significant differences in the primary endpoints of aortic root diameter and PWV, it may not have provided adequate power to detect more subtle treatment effects on carotid wall strain or cerebral blood flow velocity. This consideration is particularly relevant for carotid wall strain, which showed greater inter-animal variability than the aortic endpoints, most evident in the atorvastatin-treated female MFS group. Because the a priori power analysis was based on aortic root diameter and aortic PWV, a sample size adequate for those measures may have been insufficient to resolve a subtle effect of atorvastatin on carotid strain, particularly in females. A larger cohort would be needed to determine whether the lack of a measurable strain response reflects a true absence of effect or limited statistical power for this endpoint. The sex-dependent PCA findings, while statistically significant, should be interpreted with appropriate caution given the relatively small group sizes and the interpretive limitations of peak systolic velocity as a surrogate for cerebral perfusion. Replication in larger cohorts and with complementary measurement approaches that includes cerebrovascular resistance indices will be important for confirming these observations.

## 6. Conclusions

In summary, long-term atorvastatin treatment attenuates vascular remodeling across multiple arterial beds in the *Fbn1^C1041G/+^* mouse model of MFS, reducing aortic root dilation, lowering arterial stiffness in both the aorta and carotid artery, and normalizing carotid wall thickness. These findings demonstrate that the vascular benefits of statin therapy in MFS are not confined to the thoracic aorta and extend to structurally distinct peripheral arterial beds. At the same time, the absence of improvement in carotid wall strain and the sex-dependent responses in cerebral blood flow velocity indicate that the effects of atorvastatin are heterogeneous across vascular beds and between sexes. The divergent cerebral blood flow velocity response in male and female MFS mice, increased in females and further reduced in males, highlights the importance of sex-stratified analysis in preclinical vascular research and underscores the need for mechanistic investigation of these findings before conclusions about clinical translation can be drawn. Collectively, these results provide a rationale for further preclinical investigation of statins as adjunctive therapy for the broader vasculopathy associated with MFS, with particular attention to sex as a biological variable.

## Data Availability

The original contributions presented in this study are included in the article. Further inquiries can be directed to the corresponding author.
